# Predicting the potential habitat of bears under a changing climate in Nepal

**DOI:** 10.1007/s10661-024-13253-2

**Published:** 2024-10-23

**Authors:** Rishi Baral, Binaya Adhikari, Rajan Prasad Paudel, Rabin Kadariya, Naresh Subedi, Bed Kumar Dhakal, Michito Shimozuru, Toshio Tsubota

**Affiliations:** 1 Laboratory of Wildlife Biology and Medicine, Department of Environmental Veterinary Science, Graduate School of Veterinary Medicine, Hokkaido University, Sapporo, Japan; 2 Department of Biology, University of Kentucky, Lexington, KY USA; 3 National Trust for Nature Conservation, POB 3712, Khumaltar, Lalitpur, Nepal; 4 Department of National Parks and Wildlife Conservation, Babarmahal, Babar Mahal, Kathmandu, Nepal; 5 One Health Research Center, Hokkaido University, Sapporo, Japan

**Keywords:** Ensemble, Species distribution modeling, Occurrence points, Suitable habitat, Protected areas, Climate change

## Abstract

In Nepal, the distributions of three bear species vary: sloth bears (*Melursus ursinus*) in the lowlands, Asiatic black bears (*Ursus thibetanus*) in the mid-hills, and brown bears (Ursus arctos) in the high Himalayas. We utilized 179 occurrence points for sloth bears, 199 for Asiatic black bears, and 41 points for brown bears to construct a habitat model incorporating climate and topographic variables. Employing various species distribution modeling algorithms in BIOMOD2, the model predicts suitable habitats spanning 10,971.75 km^2^ for sloth bears; 29,470.75 km^2^ for Asiatic black bears; and 6152.97 km^2^ for brown bears. Within protected areas, the habitat for sloth bears is 4120.56 km^2^, that for Asiatic black bears is 9688.67 km^2^, and that for brown bears is 4538.67 km^2^. Chitwan National Park emerged as the prime sloth bear habitat with a core area of 918.55 km^2^ and a buffer zone of 726.485 km^2^. The Annapurna Conservation Area was deemed suitable for Asiatic black bears and brown bears, covering 2802.23 km^2^ and 2795.91 km^2^, respectively. The models projected a significant reduction in the habitat of these bear species both inside and outside protected areas. As predicted under the Shared Socioeconomic Pathways (SSP)2–4.5 scenario, sloth bears may experience 54.9% (2050) and 44.7% (2070) losses, respectively, of habitat; Asiatic black bears, 11.2% (2050) and 16.8% (2070); and brown bears, 68.41% (2050) and 82.20% (2070) losses. The overlap between sloth bears and black bears spans 38.7 km^2^, and that between brown bears and black bears is 26.6 km^2^. Notably, all three bear species exhibited suitability correlations with the intermediate temperature of the driest quarter. Examining current and projected habitats provides essential information for guiding conservation strategies and ensuring the conservation of these bear species in the face of climate change.

**Supplementary Information:**

The online version contains supplementary material available at 10.1007/s10661-024-13253-2.

## Introduction

Climate change is a substantial threat to global species conservation, emphasizing the critical need to accurately forecast its impacts on species distributions. This ability is vital for devising effective strategies for conservation and population management (Molnár et al., 2011). A particular geographic region confronted with significant risks of extinction is subject to global threats, including climate change, changes in land use, invasive species, diseases, and other human-induced impacts (Adhikari et al., 2022a, b). Successfully managing and conserving species require anticipating and understanding the implications of climate change on their distribution. Over the past decade, assessments of biodiversity vulnerability to climate change have garnered significant attention from stakeholders at various levels, including managers, planners, policymakers, and researchers (Pacifici et al., 2015; Pereira et al., 2010). Despite these challenges, understanding the impacts of climate change on biodiversity remains crucial for implementing effective conservation measures.

Among the eight global species, three species of ursine, viz. sloth bears (*M. ursinus*), Asiatic black bears (*U. thibetanus*), and brown bears (*U. arctos*), have been recorded in Nepal from lowland terai and Siwalik, mid-hill and high mountain regions, and the Himalayan regions, respectively (Chetri, 2008, 2022). These regions of Nepal Himalayas are experiencing higher rates of temperature increase and associated adverse impacts of climate change, garnering significant attention from stakeholders at various levels (Joshi et al., 2019). However, bears have received limited research and conservation attention in Nepal (Joshi et al., 1997; Kadariya et al., 2018; Paudel et al., 2022). This has hindered an adequate understanding of the impacts of climate change on bear distributions, subsequently affecting effective evidence-based conservation and population management strategies.

The sloth bear, Asiatic black bear, and brown bear in Nepal face significant conservation challenges primarily due to habitat loss, human activities, and environmental degradation. The sloth bear, endemic to the Indian subcontinent, has seen a population decline of nearly 50% over the past three decades, largely due to deforestation and agricultural expansion (Dhariya et al., 2020). In Nepal, it is globally vulnerable and nationally endangered, with its remaining population restricted to the Terai region below 2000 m, where it faces threats from habitat degradation and encroachment (Jnawali et al., 2011). The Asiatic black bear, distributed across Asia, is also endangered in Nepal, with an estimated 500 individuals residing in the mid-hills and Himalayan protected areas between 1000 and 4000 m. This species is vulnerable to poaching, habitat fragmentation, and human-wildlife conflicts related to agricultural activities (Garshelis & Noyce, 2008; Jnawali et al., 2011). Similarly, the brown bear, with a critically endangered population of fewer than 20 individuals in Nepal, is found above 3800 m and is threatened by habitat loss due to livestock grazing and infrastructure development (Aryal et al., 2010; Jnawali et al., 2011). These challenges highlight the need for focused conservation efforts across Nepal’s bear species.

Climate modeling is a vital tool used by scientists to understand historical and potential future environmental changes. To create scenarios for greenhouse gas emissions under different climate policies, scientists use shared socioeconomic pathways (SSPs). These SSPs project global socioeconomic trends until 2100 and provide different narratives about how these trends will affect climate change outcomes by the end of the century (Eyring et al., 2016; Hausfather & Peters, 2020; Riahi et al., 2017; Rogelj et al., 2018). Species distribution modeling (SDM) leverages global climate models to predict suitable habitats for species, contributing to conservation strategies and enhancing our understanding of the impact of climate change on species and communities (Baral et al., 2023; Bhandari et al., 2022; Elith & Leathwick, 2009; Guillera-Arroita et al., 2015; Sofaer et al., 2019). These models utilize environmental variables to explain present and future species distributions (Li et al., 2019; Struebig et al., 2015). By combining these approaches, scientists can make informed decisions for biodiversity conservation and anticipate the effects of changing environmental conditions on various species. Recent SDM studies often employ an “ensemble” approach, combining predictions from multiple modeling techniques for improved accuracy (Adhikari et al., 2022a; Araújo & New, 2007; Hao et al., 2019; Meller et al., 2014; Mohammadi et al., 2022). Utilizing the biomod2 package, 10 algorithms were considered for ensemble modeling. The random forest (RF), maximum entropy (Maxent), and generalized linear model (GLM) models demonstrated superior performance, with high mean true skill statistic (TSS) values (> 0.70). Consequently, RF, Maxent, and GLM were selected for their robustness in accurately predicting species distributions via species distribution modeling (SDM) (Thuiller et al., 2016).

In recent years, advancements in ecological niche–based models (ENMs) have fuelled increased research on climate change impacts (Anderson, 2013). ENMs project ecological niches based on environmental changes, with studies consistently aligning wild plant and animal range shifts with climate change predictions (Root et al., 2003; Thomas, 2010). Understanding and predicting how climate change will influence the potential habitat of these bear species are crucial for guiding conservation actions. Here, we developed a projected distribution model for three bear species by analyzing presence data and relevant bioclimatic and environmental factors. The primary objectives of this research were (a) to predict climatically suitable habitats for sloth bears, Asiatic black bears, and brown bears both at present and in the future and (b) to assess future range shifts and potential habitat overlaps among these species in response to climate change to determine the overlap. The findings from this study will play a vital role in developing a thorough conservation plan for Nepal’s bears. These insights could guide the government and conservation partners in crafting policies, planning actions, and implementing strategies aimed at ensuring the long-term survival and well-being of bear populations across the country.

## Materials and methods

### Study area

**Fig. 1:**
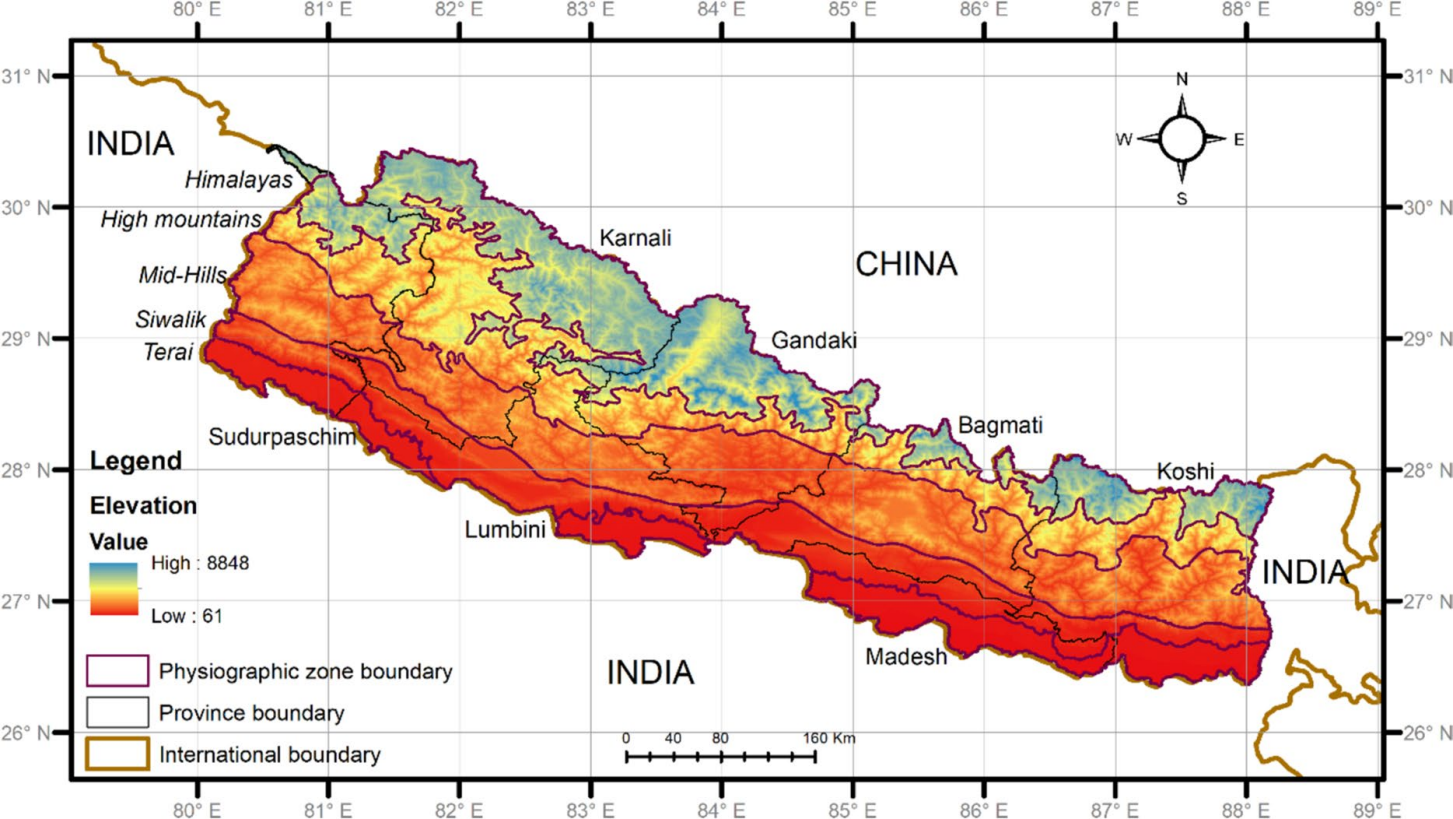
A map of the study area is presented, delineating five physiographic zones of Nepal, Terai, Siwalik, the mid-hills, the high mountains, and the Himalayas, with an elevational gradient within the region ranging from 60 to 8848 m above mean sea level

Nepal, located in the central part of the Himalayas, covers an area of 147,516 km^2^ and exhibits remarkable biodiversity due to its unique geographic location, diverse climate, and variation in elevation (Kunwar et al., 2023; Paudel et al., 2018). Its latitudinal range is approximately 26.36° N to 30.45° N, and its longitudinal range is approximately 80.06° E to 88.2° E (Fig. 1). Nepal exhibits diverse climates, spanning tropical lowlands in the southern to alpine cold semidesert conditions in the trans-Himalayan zone. The average annual rainfall ranges from 1000 to 2000 mm, with a mean estimate of 1857.6 mm. In certain lower regions, rainfall can occasionally exceed 5000 mm and is influenced significantly by altitude and topography (Bista, 2019; Ichiyanagi et al., 2007; Ohsawa et al., 1986).

The protected area network within Nepal encompasses approximately 23.3% of the total land area, including 12 national parks, one wildlife reserve, one hunting reserve, six conservation areas, and 13 buffer zones (Bhuju et al., 2007; DNPWC & DFSC, 2022). The country is divided into five broad physiographic ranges from south to north: lowland (Terai, < 300 m above sea level) covers 14% of the nation, Churia (Siwalik, 301–1000 m asl) covers 12% of the nation, middle mountain (mid-hill, 1000–3000 m asl) covers 30% of the nation, high mountain (Mahabharat, 3000–5000 m asl) covers 20% of the nation, and Himalayan (above 5000 m asl) covers 24% of the nation (Adhikari et al., 2023; DNPWC & DFSC, 2022; LRMP, 1986; Uddin et al., 2015) (Table 1). Administratively, Nepal is divided into 77 districts and seven provinces: Koshi, Madesh, Bagmati, Gandaki, Lumbini, Karnali, and Sudurpashchim.

**Table 1:** The description of the five physiographic regions in Nepal mentioning altitude, climate, vegetation type, and bear species association

Physiographic region	Elevation (m.a.s.l.)	Climate	Vegetation cover	Bear species association
Terai	Below 300 m	Tropical and sub-tropical	Dominated by *Shorea robusta* (Sal), *Acacia catechu*	Preferred by sloth bears, characterized by grasslands and dense sal forests
Siwalik (Churia/lower hills)	301–1000 m	Sub-tropical	*Shorea robusta*, *Acacia catechu*, *Alnus nepalensis*	Transitional habitat for sloth bears and some Asiatic black bears
Mid-hills (Middle Mountain)	1000–3000 m	Sub-tropical at lower altitudes, cool at higher elevations	*Alnus nepalensis*, *Castanopsis* spp., *Rhododendron* spp*.*	Habitat for Asiatic black bears, diverse vegetation ranging from sub-tropical to temperate forests
High mountain (Mahabharat)	3000–5000 m	Cold temperate	*Pinus spp.*, *Rhododendron* spp.	Preferred by Asiatic black bears and brown bears in colder, forested areas
Himalayas (High Himalayas)	Above 5000 m	Alpine to tundra	*Juniperus*–*Rhododendron* association, alpine scrub	Critical habitat for brown bears, consisting mainly of alpine meadows and barren terrain

Despite covering just 0.1% of Earth’s surface, Nepal is home to an impressive 3.2% of known flora and 1.1% of known fauna (Uddin et al., 2015). The country boasts 118 identified ecosystems supporting a rich diversity of mammalian and avian species (Adhikari et al., 2022a). Among the 212 known mammal species in Nepal, approximately 23% (49 species) are categorized as nationally threatened, including nine critically endangered, 26 endangered, 14 vulnerable, and one regionally extinct species (*Porcula salvania*). Additionally, there are seven near-threatened species and 83 species categorized as data deficient (Amin et al., 2018; Baral et al., 2019).

### Data collection and filtering

We gathered two datasets, including first-hand and second-hand occurrence records. The first-hand records were from field surveys, and the second-hand occurrence records were obtained from published research articles and unpublished government and nongovernmental reports of Nepal from 2005–2023 A.D. A total of 48 occurrence points were identified for brown bears, 252 for Asiatic black bears, and 522 for sloth bears. We compiled two datasets for sloth bears, Asiatic black bears and brown bears, integrating first-hand and second-hand occurrence records. Field surveys were conducted from September to November in 2022 and April to June in 2023 in Chitwan National Park and the Annapurna Conservation Area. In Chitwan, first-hand data for sloth bears were obtained, involving the presence of faces, footprints, and foraging traces via random walks in the forest. Secondary literature sources, including Paudel et al. (2022) and Subedi et al. (2021), supplemented our understanding of the presence of sloth bears. For Asiatic black bears, opportunistic surveys from 2022–2023 yielded 32 records from the Annapurna Conservation Area, supported by published studies by Subedi et al. (2021), Jnawali et al. (2011), Kadariya et al. (2018), and Malla et al. (2023) and reports from the National Trust for Nature Conservation (NTNC) reports (2018–2021). Similarly, brown bear datasets included 15 first-hand observations from field surveys and historical data from 2005 to 2022 sourced from Aryal et al. (2010) and Chetri (2008, 2022) and personal communications, excluding data from Aryal et al. (2012) due to reliability concerns with scat sampling. These combined datasets provided comprehensive insights into the distribution and habitat preferences of these bear species in Nepal.

The accuracy of model predictions is often influenced by the spatial autocorrelation of sampling effort in both training and test data. To address this issue and prevent sample biases and model overfitting, spatial filtering techniques were employed (Boria et al., 2014; Kramer-Schadt et al., 2013). The spatial filtering process, conducted using the SpThin package (Aiello-Lammens et al., 2015) in R software (Aryal et al., 2016; Li et al., 2019; R Core Team, 2021) (R Core Team, 2021), ensured that only one record per grid (1 × 1 km^2^) was retained, resulting in a total of 41 occurrence points for brown bears, 199 occurrence points for Asiatic black bears and 179 occurrence points for sloth bears for further habitat modeling.

### Climatic and topographic data

Nineteen bioclimatic variables and three topographic variables (slope, aspect, and elevation) (Table 2) were obtained from the WorldClim database version 2.1 (Fick & Hijmans, 2017) to predict the species distributions in the present and future. The historical data represented the average for 1970–2000, and the future scenarios considered were SSP2-4.5 (2050) and SSP2-4.5 (2070). Moreover, SSP2-4.5 has moderate and likely greenhouse gas emissions (Hausfather & Peters, 2020; Schwalm et al., 2020). To reduce multicollinearity, eight variables with a VIF < 3 were retained for species distribution modeling (Zuur et al., 2010). All the variables had a resolution of 30 arcseconds (~ 1 km^2^).

**Table 2:** Variables used to model the suitable habitats of bear species in Nepal

Bioclimatic (version 2)	Annual mean temperature	bio1	◦C
	Mean diurnal range (mean of monthly (max temp–min temp))	bio2	◦C
	Isothermality (BIO2/BIO7)	bio3	Dimensionless
	Temperature seasonality (standard deviation)	bio4	◦C
	Max temperature of warmest month	bio5	◦C
	Min temperature of coldest month	bio6	◦C
	Temperature annual range (BIO5-BIO6)	bio7	◦C
	Mean temperature of wettest quarter	bio8	◦C
	Mean temperature of driest quarter	bio9	◦C
	Mean temperature of warmest quarter	bio10	◦C
	Mean temperature of coldest quarter	bio11	◦C
	Annual precipitation	bio12	mm
	Precipitation of wettest month	bio13	mm
	Precipitation of driest month	bio14	mm
	Precipitation seasonality (coefficient of variation)	bio15	Dimensionless
	Precipitation of wettest quarter	bio16	mm
	Precipitation of driest quarter	bio17	mm
	Precipitation of warmest quarter	bio18	mm
	Precipitation of coldest quarter	bio19	mm
Topographic	Elevation	Elevation	m
	Aspect	Aspect	Degree
	Slope	Slope	Degree

Source: World Clim database version 2.1

### Species distribution modeling

In our analysis, we utilized the biomod2 package, which provides a comprehensive suite of algorithms for ensemble modeling. Specifically, first, we considered 10 different algorithms available within biomod2, namely, the random forest (RF), generalized linear model (GLM), maximum entropy (Maxent), generalized boosted model (GBM), generalized additive model (GAM), artificial neural network (ANN), multivariate adaptive regression spline (MARS), flexible discriminant analysis (FDA), surface range envelope (SRE), and generalized additive model (GAM) algorithms. Despite considering all 10 algorithms, we selected only three that demonstrated high mean true skill statistic (TSS) values (> 0.70) during model evaluation (Thuiller et al., 2016). The decision to focus on RF, Maxent, and GLM for preparing the ensemble model was based on their superior performance in accurately predicting species distributions, as indicated by the TSS metric. These algorithms have been widely recognized and extensively validated in the field of SDM because of their robustness, flexibility, and ability to handle complex relationships between environmental variables and species occurrences.

To ensure the robustness of our modeling approach, the dataset was divided into testing (20%) and training (80%) sets, with 10,000 pseudoabsence points generated for training. The criteria for generating pseudoabsence locations were based on random sampling within areas deemed environmentally unsuitable for the species following best practices in SDM (Barbet-Massin et al., 2012; Guisan et al., 2007). The selection of the testing and training data split follows common practices in SDM studies (Phillips et al., 2006). This split allows for independent validation of model performance while maximizing the use of available data for training. Specifically, the chosen ratio of 80% training data to 20% testing data strikes a balance between model training and evaluation, ensuring sufficient data for both purposes (Elith* et al., 2006).

Model performance was evaluated using area under the curve (AUC) and true skill statistics (TSS) metrics, as recommended in the literature (Thuiller et al., 2009, 2016). TSS values were used to assess the accuracy of the models, with values above 0.7 considered indicative of good model performance (Allouche et al., 2006; Thuiller et al., 2009). An ensemble model was then created using a weighted mean approach, selecting algorithms with a mean TSS > 0.7 to ensure high predictive accuracy (Marmion et al., 2009). SDM analysis was conducted using the BIOMOD2 package in R, a widely used and well-established tool for species distribution modeling (R Core Team, 2021; Thuiller et al., 2009). The resulting suitable areas predicted by the ensemble model were further analyzed by intersecting them with land use/land cover data and shape files, allowing for additional insights into habitat suitability and landscape-level conservation planning (Karra et al., 2021). The climatically suitable habitat obtained from species distribution modeling was intersected with the Sentinel-2 10-m land use/land cover data to obtain the suitable habitat within each land cover category (Karra et al., 2021).

## Results

### Contribution of variables and model performance

**Fig. 2:**
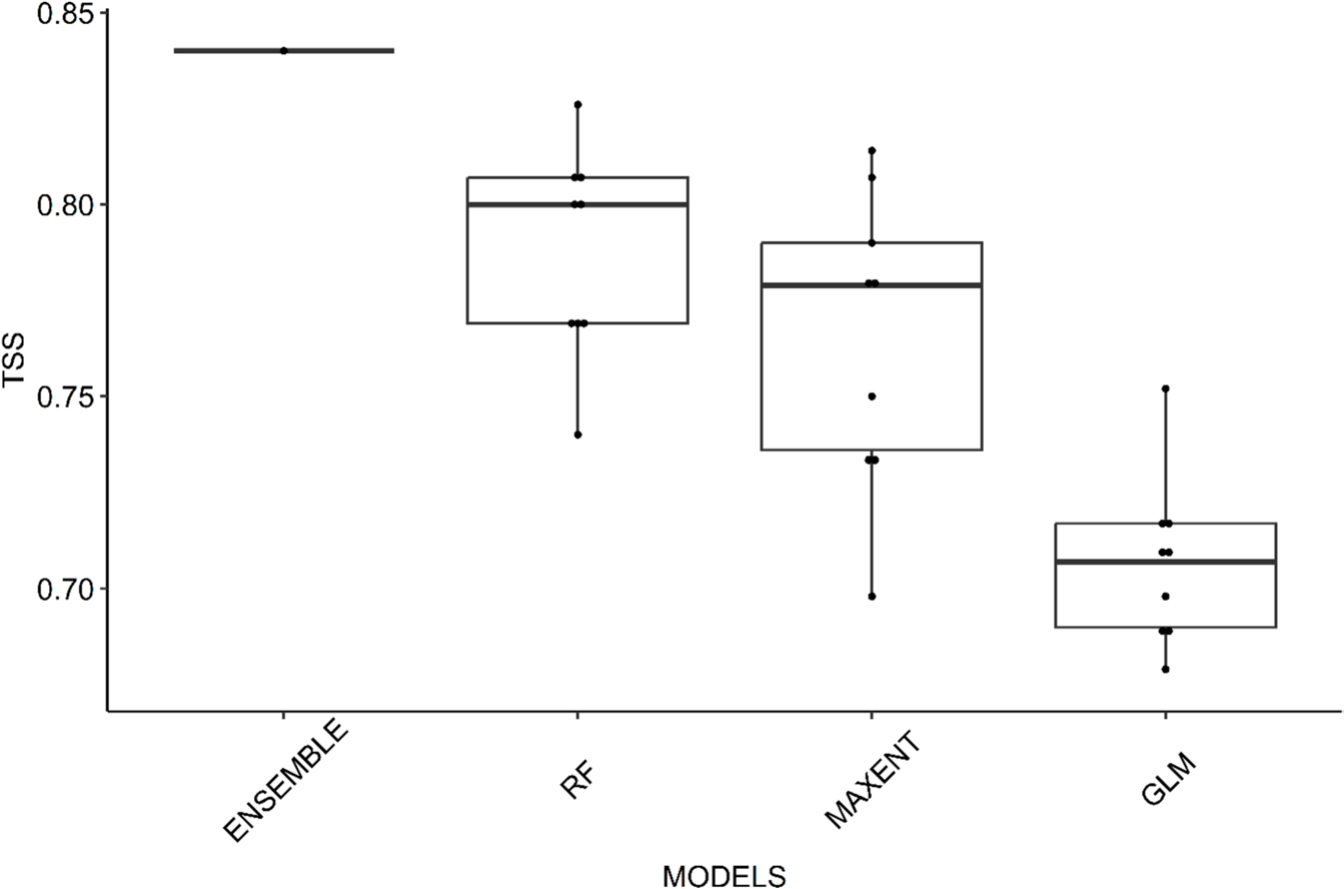
Boxplot representing the accuracy of the models used in BIOMOD2. The ensemble method yielded more accurate predictions than did the single-algorithm models: random forest (RF), maximum entropy (MAXENT), and the random generalized linear model (GLM). The upper box limit, midline, and lower box limit represent the lower quartile (Q1), the median, and the upper quartile (Q3), respectively. The whiskers represent the extension of the box to the minimum and maximum values that fall within 1.5 times the interquartile range, and any values outside this range are outliers, which are represented by red dots. TSS, true skill statistics

The suitable habitats of the three bear species were modeled with 41 occurrence points for brown bears, 199 occurrence points for Asiatic black bears, and 179 occurrence points for sloth bears. In the exploration of suitable habitats for these species through 10 SDM algorithms, three standout algorithms (GLM, MAXENT, and RF) exhibited superior predictive accuracy, each boasting an average TSS value surpassing 0.70, as depicted in Fig. 2. Similarly, the mean area under the curve (AUC) of these three algorithms was > 0.90, indicating high accuracy of the models. The ensemble model, which was generated using the three best algorithms (GLM, MAXENT, and RF), outperformed all the others, with an impressive TSS value of 0.84 (Fig. 2).

Among the eight variables employed in the SDM, bio9 (the mean temperature of the driest quarter) emerged as the paramount determinant (with the highest percentage contribution) for the ensemble model of all three species, i.e., sloth bears 35%, Asiatic black bears 57%, and brown bears 51% (Supplementary information: Fig. 5). The probability of suitable habitat for brown bears peaked at the − 5 °C mean temperature of the driest quarter; for Asiatic black bears, it peaked at 5 °C; and for sloth bears, it peaked after 15 °C (Supplementary information: Fig. 6, 7 and 8). The effects of the other variables were comparatively lower for the sloth bear and Asiatic black bear; however, for the brown bear, bio18 and bio2 also contributed significantly.

### Current distribution and suitable habitat availability of bears in Nepal

**Fig. 3:**
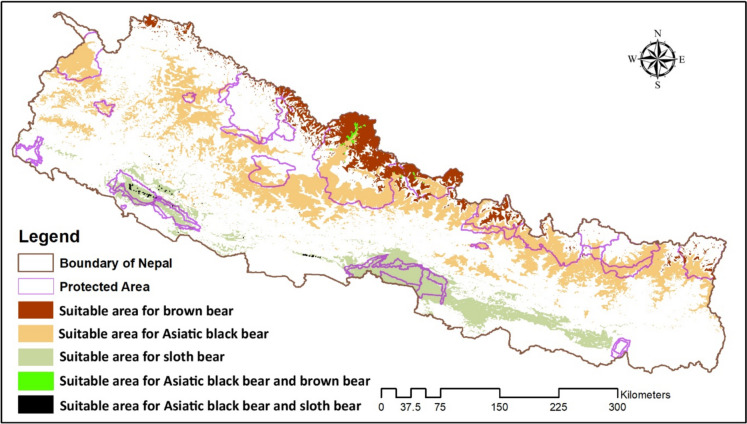
Present suitable habitat and overlaps among brown bears, Asiatic black bears, and sloth bears

Our predictive models indicated that, out of the total area of Nepal, 10,971.75 km^2^ of habitat is currently suitable for sloth bears, 29,470.75 km^2^ for Asiatic black bears, and 6152.97 km^2^ for brown bears (Fig. 3). These areas constitute 7%, 20%, and 4%, respectively, of Nepal’s total area and are current distribution for sloth bears, black bears, and brown bears. The current habitat overlap between black bears and brown bears is 0.08% and between black bears and sloth bears is 0.10%. There is no observed habitat overlap between sloth bears and brown bears (Fig. 3). Among the five physiographic regions, the Siwalik region had the most suitable habitat (9148.74 km^2^) for sloth bears, the most common mountain region (19,325.73 km^2^) for Asiatic black bears, and the Himalayas region (612.28 km^2^) for brown bears (Table 3).

**Table 3:** Present and future suitable habitat of the three bear species

Species	Year	Area	% out of total area of Nepal	Habitat (within PA)	% habitat within PA
Sloth bear	Present	10,971.75	7%	3867.57	35%
	2050	4946.32	3%	1311.47	27%
	2070	6069.23	4%	1189.25	20%
Asiatic black bear	Present	29,470.75	20%	9688.65	33%
	2050	26,162.31	18%	12,236.40	47%
	2070	24,517.31	17%	13,117.56	54%
Brown bear	Present	6152.97	4%	4538.65	74%
	2050	1943.69	1%	1813.01	93%
	2070	1106.08	1%	992.58	90%

**Fig. 4:**
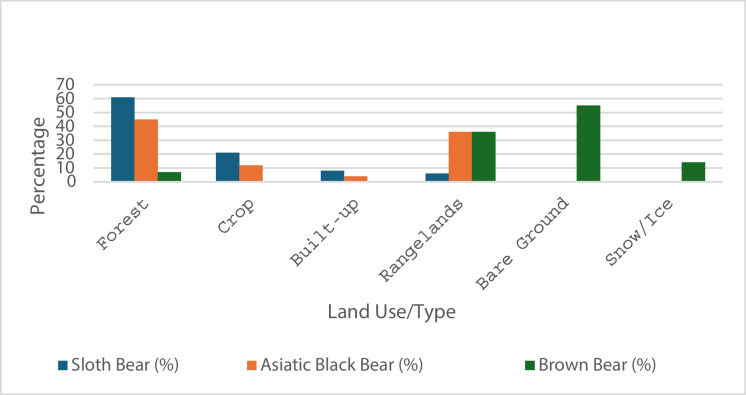
Climatically suitable habitats by land use/type for three bear species

Climatically suitable habitats constitute different proportions of land use/land cover for different bear species. For sloth bears, climatically suitable habitat constituted 61% of the forest regions, 21% of the crop, 8% of the built-up area, and 6% of the rangelands. On the other hand, 45% of the habitat of Asiatic black bears was suitable for forestland, 36% was suitable for rangelands, 12% was crop, and 4% was built-up area. For brown bears, 55% of their climatically suitable habitat consists of bare ground 36% of which consists of rangelands, 14% of which consists of snow/ice, and 7% of which consists of forest areas (Fig. 4). Moreover, our analysis revealed the provinces with the most significant suitable habitat for each bear species; Madesh Province had the most suitable habitat for sloth bears (3841.91 km^2^), Gandaki Province had the most suitable habitat for Asiatic black bears (7477.29 km^2^), and brown bears (4032.62 km2) (Fig. 4; Table 3).

Of the total suitable habitat, the sloth bear utilized only 3,867.57 km^2^ within the protected area (PA) system, with 2,497.67 km^2^ in the core area and 1369.91 km^2^ in the buffer zone. Notably, Chitwan National Park and its buffer zone accounted for the most extensive coverage, occupying 1,897.98 km^2^, followed by the Banke Bardia complex, which includes Bardiya National Park, Parsa National Park, Banke National Park, and their buffer zones (Table 4). On the other hand, the Asiatic black bear utilized 9688.63 km^2^ of the total suitable habitat within the PA system, with 8454.69 km2 in the core area and 1233.96 km^2^ in the buffer zone. Among the protected areas, the Annapurna Conservation Area had the most extensive coverage, spanning 2802.26 km^2^, followed by the Api Nampa and Gaurishankar Conservation Areas. Similarly, the brown bear incorporated a total of 4538.65 km^2^ within the PA system, comprising 4430.49 km^2^ in the core area and 108.16 km^2^ in the buffer zone. The Annapurna Conservation Area had the largest coverage of 2795.91 km^2^, followed by the Mansula Conservation Area and Langtang National Park (Table 5).

**Table 4:** Projected suitable habitats of the three bear species within each province under the current and future scenarios

Provinces	Present			2050			2070		
	Sloth bear	Asiatic black bear	Brown bear	Sloth bear	Asiatic black bear	Brown bear	Sloth bear	Asiatic black bear	Brown bear
Koshi	572.28	5723.24	257.87	152.19	5119.86	955.97	156.79	4447.17	857.87
Madhesh	3841.91	–	–	319.13	–	–	176.15	–	–
Bagmati	2794.38	5279.37	612.1	1249.23	4013.47	44.02	1467.80	4052.95	7.17
Gandaki	655.82	7477.29	4032.62	1171.57	8251.00	939.05	979.15	8485.40	241.04
Lumbini	2255.89	1901.47	-	1379.07	1528.36	-	1606.22	1430.47	
Karnali	679.07	5284.78	1250.38	474.65	5640.10	4.65	831.04	4225.13	-
Sudurpaschim	173.08	3808.03	-	201.7	1614.36	-	843.02	1882.01	-
Total	10,972.43	29,474.19	6152.97	4947.55	26,167.15	1943.69	6060.18	24,523.14	1106.08

**Table 5:** Current and projected suitable habitats for three bear species within protected areas (current, 2050, and 2070)

Protected area		Present						2050						2070					
		Sloth bear		Asiatic black bear		Brown bear		Sloth bear		Black bear		Brown bear		Sloth bears		Black Bear		Brown Bear	
Conservation area (CA)		Core area	Buffer zone	Core area	Buffer zone	Core area	Buffer zone	Core area	Buffer zone	Core area	Buffer zone	Core area	Buffer zone	Core area	Buffer zone	Core area	Buffer zone	Core area	Buffer zone
1	Annapurna CA	–	–	2802.26	–	2795.91	–			3,803.78		724.57				4026.04		214	
2	Kangchenjunga CA	–	–	453.41	–	161.84	–			408.83		694.46				382.25		716.85	
3	Manaslu CA	–	–	430.71	–	818.46	–			947.01		214.38				1169.28		32.33	
4	Gaurishankar CA	–	–	1271.19	–	68	–			1511.47		7.75				1547.70		2.04	
5	Api Nampa CA	–	–	1273.67	–	–	–			525.6						679			
National parks (NPs)																			
6	Shey Phoksundo NP	–	–	83.55	215.13	137.13	104.75			213.45	329.57					211.16	295.75		
7	Langtang NP	–	–	489.11	371.08	440.7				1186.67	144.53	33.53				1480.30	107.64	5.17	
8	Sagarmatha NP	–	–	90.05	20.41	3.27	3.41			231.26	166.01	108.63	0.52			194.22	399.53	12.47	
9	Shivapuri NP	–	–	78.65	–	–	–			4.42						0.52			
10	Chitwan NP	918.55	726.48	–	–	–	–	504.25	106.25					373.13	27.08				
11	Makalu Barun NP	–	–	653.11	526.83	5.19				958.79	491.37	28.66				976.34	369.5	9.72	
12	Rara NP	–	–	9.72	–	–	–			76.67						59.1			
13	Khaptad NP	–	–	147.54	–					86.98						87.87			
14	Banke NP	374.41	128.46	–	0.52	–	–	84.73	55.63			–	–	65.68	49.54				
15	Bardia NP	621.51	250.85	25.67	–	–	–	266.77	22.99					263.78	132.66				
16	Parsa NP	576.32	258.36	–	–	–	–	152.36	118.49					142.44	129.61				
17	Suklaphanta NP	5.81	4.71	–	–	–	–								5.47				
Hunting reserve (HR)																			
18	Dhorpatan HR	–	–	576.05	–	–	–			1149.99						1131.38			
Wildlife reserve (WR)																			
19	Koshi Tappu WR	1.06	1.06	–	–	–	–												
Total		**2497.67**	**1369.91**	**8454.70**	**1233.96**	**4430.49**	**108.16**	**1008.11**	**303.37**	**11,104.93**	**1131.48**	**1811.97**	**0.52**	**845.02**	**344.35**	**11,945.15**	**1172.42**	**992.58**	
Grand total		**3867.57**		**9688.65**		**4538.65**		**1311.48**		**12,236.40**		**1812.48**		**1189.37**		**13,117.57**		**992.58**	

### Future range shift

The future range of suitable habitats for bear species in Nepal is expected to undergo significant changes and reductions. Under the SSP2-4.5 scenario, 54.92% and 44.69% of the current suitable habitat for sloth bears are predicted to be lost by 2050 and 2070, respectively. Similarly, 11.23% and 16.81% of the suitable habitat for Asiatic black bears and 68.41% and 82.20% of the suitable habitat for brown bears are predicted to be lost by 2050 and 2070, respectively (Table 5). By the years 2050 and 2070, sloth bears are projected to constitute only 3% and 4%, respectively, of Nepal as suitable habitat, Asiatic black bears will decrease from 18 to 17%, and brown bears will constitute only 1% of Nepal’s total area as suitable habitat by 2070.

The area of suitable habitat within protected areas is also expected to change. For sloth bears, suitable habitat within the current boundaries of PAs is predicted to decrease from 35 to 27% in 2050 and from 20% in 2070. Conversely, for Asiatic black bears, suitable habitat inside PAs is predicted to increase from 33 to 47% in 2050 and significantly to 54% in 2070. Similarly, for brown bears, the prevalence of this pest will increase within protected areas from 74 to 90% in 2050 and 93% in 2070 (Table 3).

**Fig. 5:**
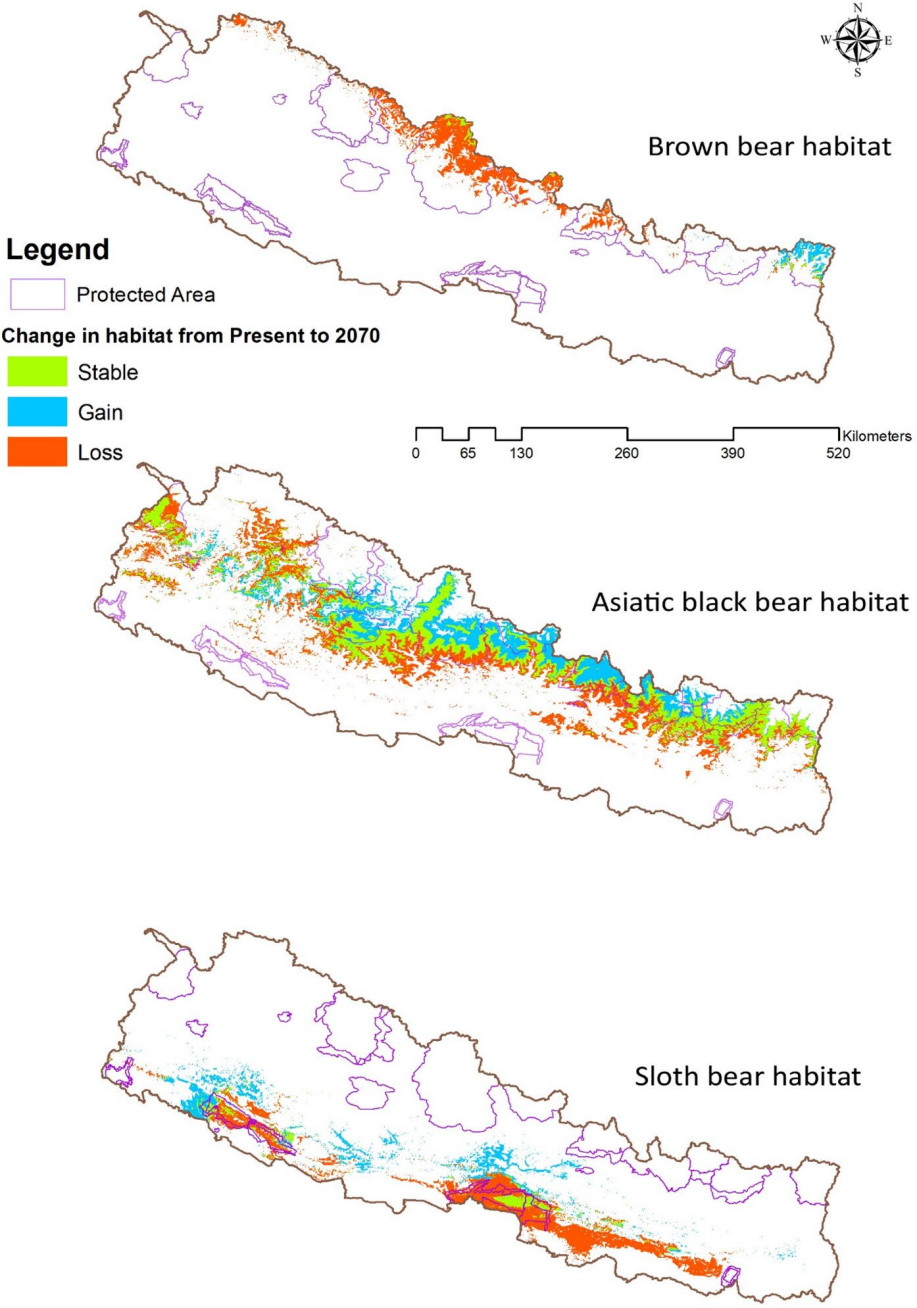
Projected changes in suitable habitat for three bear species (from present to 2070) under the SSP2-4.5 scenario, where green represents stable habitat, blue represents gain, and orange represents loss SSP (shared socioeconomic pathway)

In Table 5, the stable, gain, and loss of potential habitat were delineated for the years 2050 and 2070, respectively. According to the model, the brown bear exhibited potential habitat gain primarily within the protected areas of the eastern part of Nepal, notably within the Kanchenjunga Conservation Area. Conversely, significant potential habitat loss was observed in the Himalayan range, while stable habitat ratios were noted within the protected areas of the Annapurna and Kanchenjunga conservation areas (Fig. 5).

In contrast, the Asiatic black bears showcased potential habitat gains both within and outside the protected areas of the Himalayan ranges, with stability observed in certain high mountain ranges, particularly outside protected areas. However, a notable decline in potential habitat was observed in the mid-hill regions for this species (Fig. 5). Similarly, for the sloth bears, potential habitat gains were observed predominantly outside the protected areas in the middle and western landscapes. Stable habitat conditions for sloth bears were identified within protected areas such as Chitwan National Park and Bardia National Park, as well as nearby buffer zones in the Terai region. Conversely, substantial habitat loss was documented in the Terai landscape, spanning from the western to eastern regions of Nepal, particularly outside protected areas (Fig. 5).

### Potential habitats overlapping with future climatic conditions

The potential overlap of habitats among these three species was found to be low. Limited habitat overlap was observed between the sloth bear and Asiatic black bear, as well as between the black bear and brown bear. Specifically, the overlap between the sloth bear and the Asiatic black bear was 38.68 km^2^, while it was 26.85 km^2^ between the Asiatic black bear and the brown bear. However, no habitat overlap was found between the sloth bears and brown bears (Fig. 3).

Habitat overlap for sloth bears and black bears was most prominent along the northern parts of Bardia National Park and the Siwalik hills between Chitwan National Park and Banke National Park (Fig. 3). Additionally, in the Annapurna and Manaslu Conservation areas, the habitats were predicted to be suitable for both Asiatic black bears and brown bears. However, the extent of overlap is predicted to decrease to 5.95 km^2^ in 2050 and 6.88 km^2^ in 2070 between sloth and black bears, while there will be an increase in Asiatic black bear and brown bear overlap of 27.26 to 30.55 km^2^ in 2050 and 2070, respectively (Table 5).

## Discussion

Our study utilized ecological niche modeling (ENM) to assess the distribution of sloth bears, Asiatic black bears, and brown bears under current and future climate scenarios in Nepal. ENM has been widely recognized as an effective tool for predicting species distributions, particularly in the context of climate change and habitat suitability (Ashrafzadeh et al., 2022; Rezaei et al., 2022). The high true skill statistic (TSS) value (0.84) indicates that our ensemble model provided accurate predictions of potentially suitable habitats for these species. This approach aligns with similar studies that have successfully used predictive modeling for large carnivore conservation planning (Duan et al., 2014; Schadt et al., 2002; Su et al., 2018).

### Current habitat distribution

The predicted habitats for the three bear species highlight their largely distinct distributions across Nepal. For sloth bears, our model indicates that 7% of Nepal’s total land area, primarily located in the Siwalik and Terai regions, serves as a suitable habitat. These regions are predominantly covered by forests (61%), followed by cropland (21%), built-up areas (8%), and rangelands (6%). While previous research from radio collaring of 18 individual and 17 individuals in the 1990s has shown that sloth bears are highly adaptable to various habitats, including dry or moist forests, savannahs, and grasslands (Garshelis et al., 1999; Joshi et al., 1995), our findings emphasize the critical importance of forested habitats at the landscape level. Although grasslands provide essential food resources on a finer scale (Paudel et al., 2022), forests remain the primary habitat type supporting sloth bear populations.

For Asiatic black bears, approximately 20% of Nepal’s land area is identified as suitable habitat, primarily situated in the high and middle mountain regions. Asiatic black bears are known to prefer temperate forests (Chetri, 2013; Kadariya et al., 2018; Subedi et al., 2021), and our results align with this, showing that 45% of their suitable habitat consists of forests. Additionally, 36% of their habitat comprises rangelands, followed by cropland (12%) and built-up areas (4%). These results are consistent with their wider distribution across Asia, where they are found in a range of habitats, including forests and rangelands (Garshelis & Steinmetz, 2020). In Pakistan, Asiatic black bears inhabit mountainous areas within the Himalayas, characterized by complex ecosystems, steep slopes, rugged terrains, and harsh weather conditions (Zahoor et al., 2022). This distribution pattern is similar to that observed in Nepal, underscoring the species’ adaptation to diverse and challenging environments.

In contrast, brown bears have much more limited habitat availability in Nepal, with only 4% of the total land area considered suitable for them. These habitats are predominantly restricted to the high Himalayas, aligning with previous studies that identify alpine meadows and grasslands as key habitats for brown bears (Aryal, 2012; Wu, 2014). The prevalence of bare ground and rangelands in their suitable habitats underscores the urgent need for the conservation of these fragile ecosystems to ensure the long-term survival of brown bear populations in the region.

### Suitability of habitats within protected areas (PAs)

The study identifies key protected areas (PAs) for bear species conservation, with notable habitats for sloth bears in Madesh Province and for Asiatic black bears and brown bears in Gandaki Province. Chitwan National Park provides the largest area of suitable habitat for sloth bears, reinforcing its critical role in the species’ conservation (Paudel et al., 2022; Sharma et al., 2022). Similarly, the Annapurna Conservation Area holds substantial habitats for both Asiatic black bears and brown bears (Chetri, 2008; Kadariya et al., 2018).

However, recent research indicates that significant bear habitats extend beyond these protected areas (Malla et al., 2023; Mohammadi et al., 2021; Sharma et al., 2022). Nepal boasts an extensive network of protected areas, particularly in the Himalayas and high mountain physiographic ranges; these areas serve as crucial corridors facilitating the movement of wildlife, including prominent species such as black bears and brown bears (Panthi et al., 2019a; Zahoor et al., 2021a). This highlights the need for a comprehensive conservation strategy that goes beyond PAs, incorporating landscape-level connectivity to prevent habitat fragmentation. Enhancing corridors between existing PAs and integrating additional zones into the conservation framework could significantly improve habitat protection for bears in Nepal, especially in light of ongoing environmental changes (Brennan et al., 2022). Furthermore, engaging local communities in conservation initiatives is essential. Collaborative approaches that combine traditional knowledge with scientific research are key to the long-term sustainability of bear populations, ensuring that both formal and informal landscapes contribute to their conservation. These efforts are vital for addressing habitat fragmentation and enhancing the effectiveness of conservation programs across Nepal’s diverse ecosystems.

### Habitat overlap among bear species

The potential for habitat overlaps between sloth bears and Asiatic black bears is notably evident in Nepal’s western Terai region. Our model predicts patches of overlapping habitats, particularly in the northern parts of Bardiya National Park and areas between Banke and Chitwan National Parks. This cooccurrence has been documented in the Babai Valley of Bardiya National Park and extends into northern India, where both species share tropical forest and grassland ecosystems along the Gangetic floodplains and Siwalik Mountains, rich in biodiversity and prey availability (Joshi et al., 1997; Yadav et al., 2017).

Similar overlaps have been observed between sun bears and black bears in Southeast Asia, with dietary specialization, such as the insectivorous diet of sun bears, facilitating coexistence (Steinmetz et al., 2013). In Nepal, sloth bears exhibit a similar specialization in consuming ants and termites, which may reduce direct competition with black bears, whose diet focuses more on plant materials (Bhandari et al., 2022; Garshelis et al., 1999; Kadariya et al., 2018; Rai et al., 2022). This differentiation in feeding habits likely plays a key role in mitigating competition and enabling the coexistence of these species in overlapping habitats. This pattern of habitat and dietary overlap is also observed in North-East India, where these species co-occur and demonstrate resource partitioning (Garshelis et al., 2022).

Lowland Terai habitats, which support a diverse range of mammals from the Felidae, Canidae, and Viverridae families, further compound competition for resources. However, sloth bears’ insectivorous diet helps minimize direct dietary overlap with other large mammals, as noted by Sharma et al. (2023). On the other hand, black bears, which rely more on vegetation, may face higher competition for plant-based resources. This competition is mitigated by the differences in habitat use, with sloth bears generally occupying lower elevations due to their dependence on termite and ant populations, which decline at higher altitudes (Gathorne-Hardy & Eggleton, 2001).

In high-elevation regions, Asiatic black bears are more likely to share habitats with brown bears. Elevation, land use, and distance to water are significant factors influencing the distribution of Asiatic black bears, while brown bears are predominantly restricted to high mountain areas due to their diet of small mammals like marmots and unfavorable agricultural conditions at those altitudes (Aryal et al., 2012; Rai et al., 2022). This dietary specialization of brown bears and their preference for harsher, higher-elevation environments limits significant overlap with Asiatic black bears, despite the shared habitat. These findings highlight the complexity of habitat use among bear species in Nepal and underscore the importance of understanding dietary preferences and habitat specialization to inform effective conservation strategies. A landscape-level conservation approach, incorporating both protected areas and the broader ecosystems that support these species, is crucial for mitigating potential competition and ensuring the long-term survival of bears in Nepal’s diverse landscapes.

### Future range shifts due to climate change

Our findings indicate significant impacts of climate change on bear species’ habitats in Nepal. The model predicts a decline in suitable habitats across all five landscapes, mirroring trends observed in other studies (Ashrafzadeh et al., 2022; Dar et al., 2021; Penteriani et al., 2019; Rai et al., 2022; Su et al., 2015; Zahoor et al., 2021b). By 2050, under moderate emission scenarios, sloth bears, Asiatic black bears, and brown bears are expected to see significant habitat reductions, with only 3%, 18%, and 1% of their respective habitats remaining. This highlights the vulnerability of these species to climate-induced habitat loss.

The study further reveals that while sloth bear habitats within protected areas are predicted to shrink, Asiatic black bears and brown bears could experience habitat expansion. This underscores the critical role of protected areas and the necessity for enhanced habitat connectivity to mitigate fragmentation. However, comprehensive conservation strategies that include corridors and non-protected areas are vital to ensure the long-term survival of these species (Zahoor et al., 2021b).

Nepal’s fluctuating forest cover, from 45% in 1964 to 40% in 2015 (Chapagain & Aase, 2020), presents both challenges and opportunities for conservation. Increased forest governance has boosted forest regeneration, yet the future survival of bear species depends on expanding connectivity and reducing habitat fragmentation outside current protected areas (Paudel et al., 2022). Effective conservation planning must transcend national borders, as bear habitats are not confined to Nepal. The Hindu Kush Himalayan (HKH) region, spanning Afghanistan to Myanmar, is critical for biodiversity conservation, and ICIMOD’s transboundary initiatives, such as the Kangchenjunga Landscape Initiative, aim to maintain connectivity and foster international collaboration for species protection. Conservation efforts must focus on adaptive management, habitat restoration, and engaging local communities to safeguard bear populations in changing environments.

Asiatic black bears’ preference for riparian habitats and their extensive movement beyond protected areas demand strategies that upgrade reserves and enhance corridors (Krosby et al., 2016). Furthermore, brown bears may shift to higher altitudes due to climate change, similar to shifts seen in Asiatic black bears, necessitating a multi-species conservation approach (Ashrafzadeh et al., 2022). This emphasizes the importance of integrating habitat suitability and connectivity models into national conservation strategies, especially for large carnivores (Kaszta et al., 2020; Liu et al., 2018).

In conducting habitat modeling for bear species in Nepal using ensemble techniques, several limitations and recommendations have emerged. The model utilized 19 bioclimatic and three topographic variables but incorporating more variables could improve accuracy and provide different outcomes. Limited data on brown bears, partly due to their low population and migratory nature from the Tibetan Plateau, poses a challenge for precise habitat modeling so the number of occurrence points should be searched more in the future by intensive survey. Additionally, presence data were collected in only two seasons; future studies should incorporate data from all seasons for better results.

The lack of detailed environmental data across Nepal restricts the accuracy of the model and its transferability to other regions. To overcome this, finer-scale data collection and integrating local knowledge into the modeling process are recommended (Bista et al., 2018). Continuous monitoring and validation with independent datasets are also essential to ensure reliable predictions. Furthermore, engaging stakeholders, including local communities, is critical for adaptive conservation strategies that support bear conservation in Nepal (Panthi et al., 2019b).

### Conclusion and implication on conservation

The populations of sloth bears, Asiatic black bears, and brown bears in Nepal are expected to experience substantial distribution shifts and habitat reductions due to climate change. Our ensemble modeling approach, integrating climatic and topographic data, highlights the vulnerability of these species. While our study provides valuable predictions for future suitable habitats, it is crucial to recognize the complex interactions between climate change and other anthropogenic factors, such as land use changes, agricultural expansion, and habitat fragmentation, which may further limit the availability of suitable habitats.

Protected areas play an essential role in bear conservation in Nepal, but they may not be sufficient on their own to mitigate future challenges posed by climate change. In addition to establishing ecological corridors, adaptive management strategies must be implemented to address changes in land use practices, human-wildlife conflict, and development pressures. Further research is needed to assess how factors like land cover changes, deforestation, and infrastructure development might influence the actual availability and quality of habitats in the future. Incorporating such factors into conservation planning will enhance the resilience of bear populations in Nepal and safeguard their long-term survival.

## Supplementary Information

Below is the link to the electronic supplementary material.

Supplementary file1 (DOCX 464 KB)

## Data Availability

No datasets were generated or analysed during the current study.
