# Correction to: Predicting the potential habitat of bears under a changing climate in Nepal

**DOI:** 10.1007/s10661-024-13365-9

**Published:** 2024-11-21

**Authors:** Rishi Baral, Binaya Adhikari, Rajan Prasad Paudel, Rabin Kadariya, Naresh Subedi, Bed Kumar Dhakal, Michito Shimozuru, Toshio Tsubota

**Affiliations:** 1 Laboratory of Wildlife Biology and Medicine, Department of Environmental Veterinary Science, Graduate School of Veterinary Medicine, Hokkaido University, Sapporo, Japan; 2 Department of Biology, University of Kentucky, Lexington, KY USA; 3 National Trust for Nature Conservation, Khumaltar, POB 3712, Lalitpur, Nepal; 4 Department of National Parks and Wildlife Conservation, Babarmahal, Babar Mahal, Kathmandu, Nepal; 5 One Health Research Center, Hokkaido University, Sapporo, Japan

**Correction to: Environ Monit Assess (2024) 196:1097**

10.1007/s10661-024-13253-2

The article Predicting the potential habitat of bears under a changing climate in Nepal, written by Rishi Baral, Binaya Adhikari, Rajan Prasad Paudel, Rabin Kadariya, Naresh Subedi, Bed Kumar Dhakal, Michito Shimozuru and Toshio Tsubota, was originally published Online First without Open Access. After publication in volume 196, issue 11, article ID 1097 the author decided to opt for Open Choice and to make the article an Open Access publication. Therefore, the copyright of the article has been changed to © The Author(s) 2024 and the article is forthwith distributed under the terms of the Creative Commons Attribution 4.0 International License, which permits use, sharing, adaptation, distribution and reproduction in any medium or format, as long as you give appropriate credit to the original author(s) and the source, provide a link to the Creative Commons licence, and indicate if changes were made. The images or other third party material in this article are included in the article’s Creative Commons licence, unless indicated otherwise in a credit line to the material. If material is not included in the article’s Creative Commons licence and your intended use is not permitted by statutory regulation or exceeds the permitted use, you will need to obtain permission directly from the copyright holder. To view a copy of this licence, visit http://creativecommons.org/licenses/by/4.0/.

