# Supplementary material for: Predicting the potential habitat of bears under a changing climate in Nepal
Source: Environ Monit Assess. 2024 Oct 23;196(11):1097. doi: 10.1007/s10661-024-13253-2 (PMC11549196; doi:10.1007/s10661-024-13253-2)
Supplement: Supplementary file 1 — Supplementary file1 (DOCX 464 KB) [file 10661_2024_13253_MOESM1_ESM.docx]

**Predicting the potential habitat of bears under a changing climate in Nepal**

**Rishi Baral^1^, Binaya Adhikari ^2^, Rajan Prasad Paudel ^3^, Rabin Kadariya^3^, Naresh Subedi^3^, Bed Kumar Dhakal^4^, Michito Shimozuru^1,5^, Toshio Tsubota^1^**

^1^ Laboratory of Wildlife Biology and Medicine, Department of Environmental Veterinary Science, Graduate School of Veterinary Medicine, Hokkaido University, Sapporo, Japan

^2^Department of Biology, University of Kentucky, Lexington, KY, USA

^3^National Trust for Nature Conservation, Khumaltar, Lalitpur, POB 3712, Nepal

^4^ Department of National Parks and Wildlife Conservation, Babarmahal, Babar Mahal, Kathmandu, Nepal

^5^ One Health Research Center, Hokkaido University, Sapporo, Japan

**Corresponding email**: right.rishi1@gmail.com and tsubota@vetmed.hokudai.ac.jp

**Supplementary information**


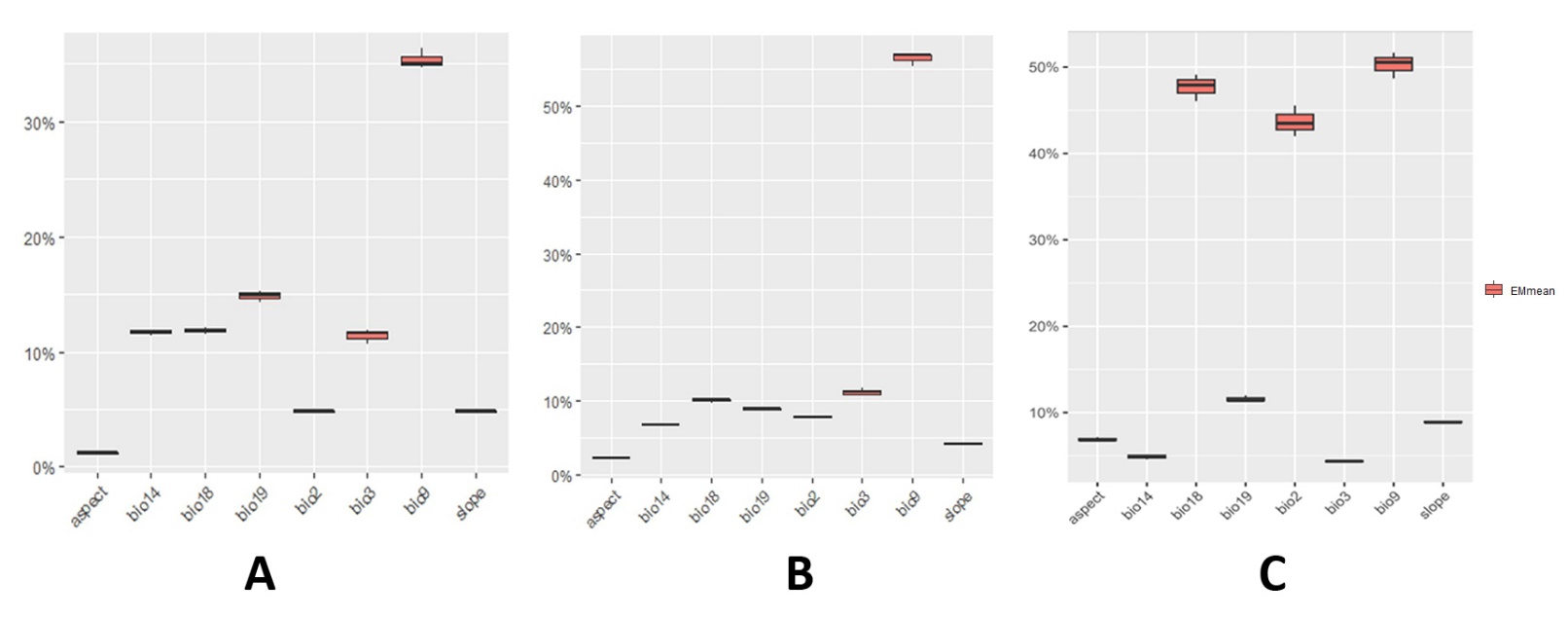


**Fig. 5**: Box plot presenting the mean percentage contribution of each variable for building the ensemble model. Plot A represents sloth bear, B represents Asiatic black bear, and C represents brown bear. X axis presents topographic/bio-climatic variables aspect, bio14, bio18, bio19, bio2, bio3, bio9 and slope respectively, whereas Y axis presents the percentage contribution of variables to build model.


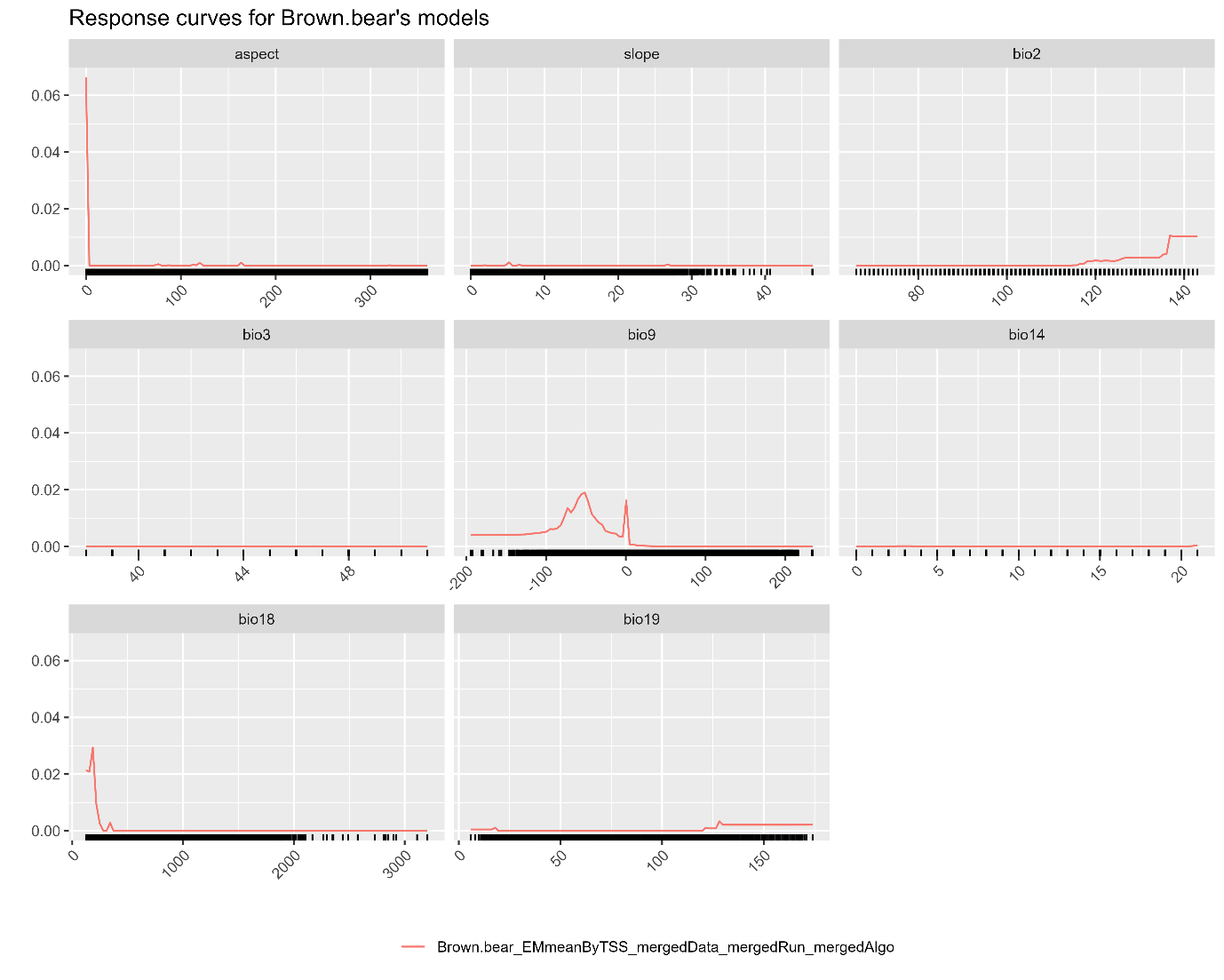


**Fig. 6**: Response curves of brown bear’s (Red line) to different bio-climatic variables viz. aspect, slope, bio2, bio3, bio9, bio14, bio18, and bio19, respectively, from top left to bottom right, respectively.


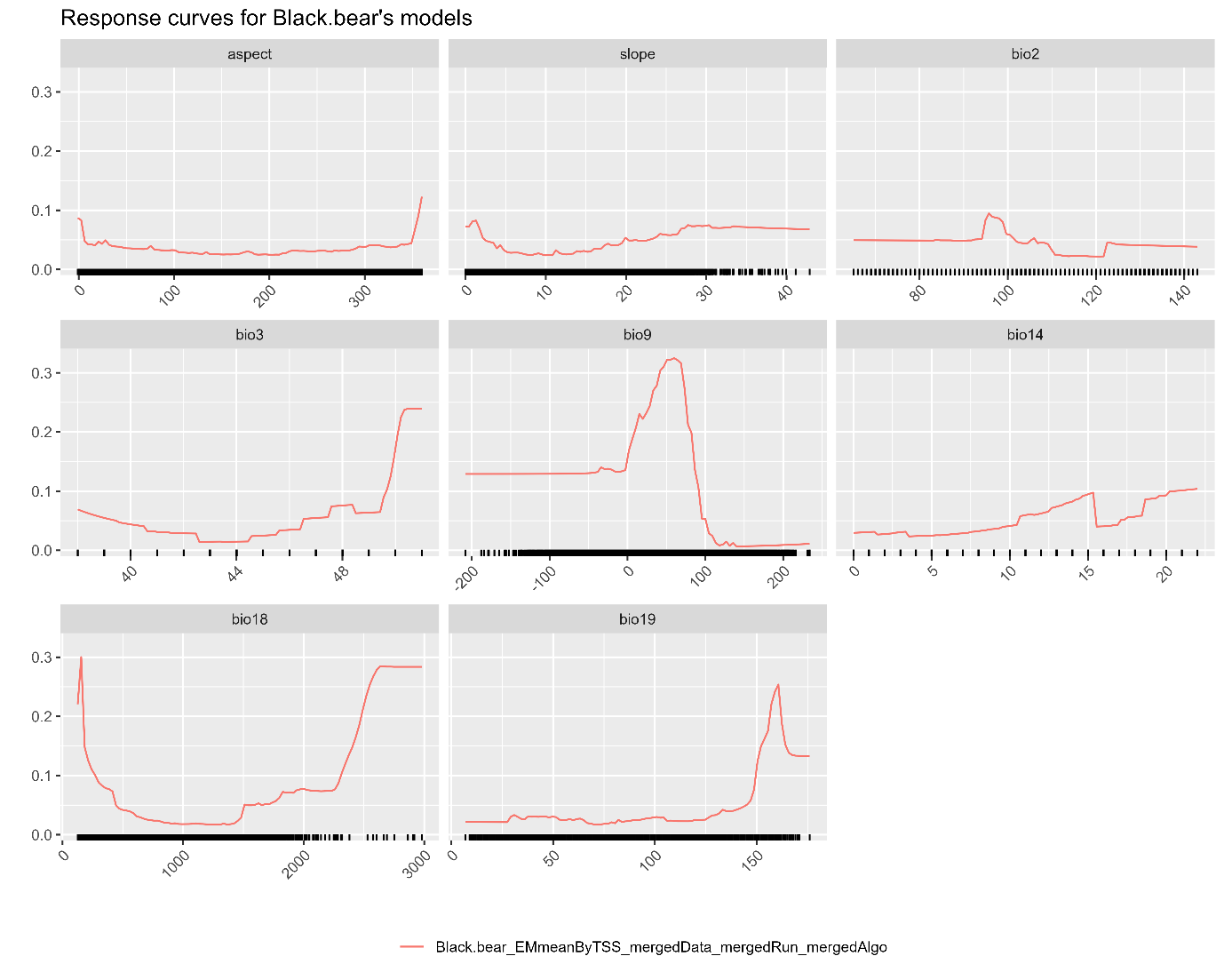


**Fig 7:** Response curves of Asiatic black bear’s (Red line) to different bio-climatic variables viz. aspect, slope, bio2, bio3, bio9, bio14, bio18, and bio19, respectively, from top left to bottom right, respectively.


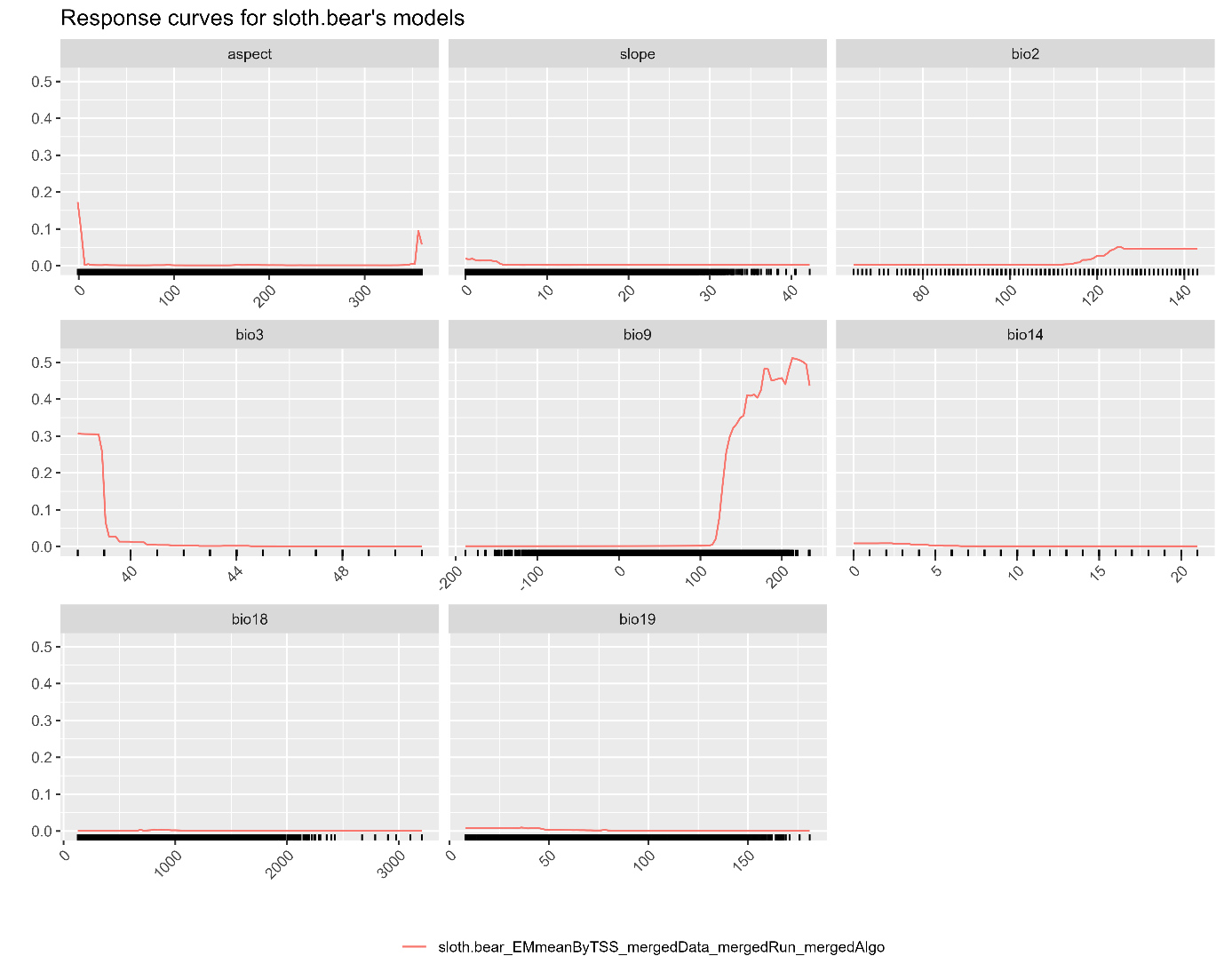


**Fig. 8:** Response curves of sloth bears (Red line) to different bio-climatic variables viz. aspect, slope, bio2, bio3, bio9, bio14, bio18, and bio19, respectively, from top left to bottom right, respectively.
